# Perineural spread of malignant head and neck tumors: review of the literature and analysis of cases treated at a teaching hospital

**DOI:** 10.1590/0100-3984.2015.0215

**Published:** 2017

**Authors:** Mauro César Silveira Moreira, Antonio Carlos dos Santos, Murilo Bicudo Cintra

**Affiliations:** 1 MSc, Radiologist at the Hospital das Clínicas da Faculdade de Medicina de Ribeirão Preto da Universidade de São Paulo (HCFMRP-USP), Ribeirão Preto, SP, Brazil.; 2 PhD, Professor, Head of the Center for Imaging Sciences and Medical Physics of the Hospital das Clínicas da Faculdade de Medicina de Ribeirão Preto da Universidade de São Paulo (HCFMRP-USP), Ribeirão Preto, SP, Brazil.; 3 PhD, Radiologist at the Hospital das Clínicas da Faculdade de Medicina de Ribeirão Preto da Universidade de São Paulo (HCFMRP-USP), Ribeirão Preto, SP, Brazil.

**Keywords:** Head and neck neoplasms/diagnosis, Diagnostic imaging, Tomography, X-ray computed, Magnetic resonance imaging, Neoplasias de cabeça e pescoço/diagnóstico, Diagnóstico por imagem, Tomografia computadorizada, Ressonância magnética

## Abstract

Perineural tumor spread refers to the migration of tumor cells along nerve tissues. It worsens the prognosis, increases the recurrence rate, and diminishes 5-year survival by up to 30%. It is an important finding on imaging tests employed in the staging of patients with head and neck cancers, because it cannot be assessed by the surgeon alone. Nevertheless, it is frequently overlooked. In this study, we reviewed the literature regarding the imaging and pathophysiological aspects of this type of dissemination. We also analyzed ten imaging tests, obtained from a teaching hospital in Brazil, in which there were radiological signs of perineural tumor spread.

## Abstract (pt)

Disseminação perineural de tumores refere-se à extensão de células tumorais ao longo do tecido dos nervos. Acarreta um pior prognóstico, aumenta a taxa de recorrência e diminui a expectativa de vida em cinco anos em até 30%. É um achado importante em exames de imagem quando se estadia pacientes com cânceres de cabeça e pescoço, já que não pode ser avaliado somente pelo cirurgião. Todavia, é uma alteração que frequentemente passa despercebida. No presente estudo, revisamos trabalhos científicos da literatura médica sobre os achados de imagem e os aspectos fisiopatológicos desse tipo de disseminação e analisamos 10 exames de imagem com sinais radiológicos de disseminação perineural oriundos de um hospital-escola brasileiro.

## INTRODUCTION

Perineural spread (PNS) of a tumor refers to the migration of tumor cells along nerve tissues, a finding that is related to a worse prognosis. It is a process that can occur in various parts of the body, not only in benign conditions (infections, granulomatous diseases, and benign tumors) but also in malignant diseases, and can have retrograde or anterograde effects on the nerve. In this paper, we focus only on the spread of head and neck malignancies. This pattern of tumor spread can be overlooked clinically and in imaging studies, tumor cells outside of the surgical/radiotherapy site thus being protected. If PNS is not detected or its extent is underestimated by imaging methods, the treat­ment probably will not control the disease. Patients in whom there is PNS have a three times greater risk of regional recurrence and a 30% lower 5-year survival rate^(1)^.

It is crucial for radiologists to detect PNS, because, unlike other characteristics such as the location of the tumor, its approximate size, and the presence of lymph node disease, it is a parameter that cannot be evaluated by the surgeon during clinical examination. In a study conducted by Lee et al.^(2)^, involving 38 patients in whom there was histopathological evidence of PNS of neoplasms of the head and neck, only 13.1% of the radiology reports mentioned the possibility of this type of dissemination, whereas a retrospective study of those same cases revealed signs of PNS in 78.9% of the patients.

A detailed understanding of the anatomy of the cranial nerves, as well as that of the head and neck, is of paramount importance for the radiological assessment of PNS. One should evaluate each potentially damaged nerve in its full extent, from its terminus (the peripheral site) to its origin (in the central nervous system). Although PNS appears continuous on microscopy, it can have a discontinuous appearance on imaging, especially in regions near the foramina at the base of the skull.

The symptoms that most often accompany PNS are pain, a burning sensation, and numbness at the site of innervation, any of which can appear years after the initiation of therapy, although up to 40% of patients are asymptomatic^(3)^. Other possible symptoms include weakness and denervation of the masticatory muscles, as well as facial paralysis. A diagnosis of “Bell’s palsy”, or idiopathic facial paralysis, should not be made  if the symptoms persist. In such cases, careful radiological evaluation should be performed in order to exclude mass lesions.

In terms of absolute numbers, the tumor most often associated with PNS is squamous cell carcinoma, followed by adenoid cystic carcinoma, basocellular carcinoma, lymphoma, and desmoplastic melanoma. However, despite its rarity, adenoid cystic carcinoma has the greatest propensity for PNS.

In the present study, we review the literature regarding PNS of head and neck cancer, emphasizing the radiological aspects, as well as presenting an overview of the pathophysiological and clinical aspects of the condition. We also analyzed ten cases of patients treated at the Hospital das Clínicas da Faculdade de Medicina de Ribeirão Preto da Universidade de São Paulo (HCFMRP-USP), in the city of Ribeirão Preto, SP, Brazil, all of whom presented PNS on imaging studies.

## PATHOPHYSIOLOGICAL CONSIDERATIONS

The histopathological definition of PNS remains controversial. Batsakis^(4)^ described neurotropism as tumor invasion “in, around, and through the nerve”. Liebig et al.^(5)^, although agreeing with that description, added that PNS is also characterized by tumors in close proximity to the nerve sheath and involving at least 33% of its circumference or by tumor cells within any of the three layers of the neural sheath. Dunn et al.^(6)^ defined PNS simply as the presence of tumor cells in the perineural space.

The mechanism that leads to PNS is still not completely understood. In a study conducted by Vural et al.^(7)^, PNS was found to correlate positively with the expression of neural cell adhesion molecules and membrane glycoproteins that mediate cell adhesion. However, the authors found that expression of neural cell adhesion molecules did not correlate with local recurrence, tumor differentiation, lymph node involvement, or a disease-free interval. In most cases, PNS is contiguous. In a study of 51 patients, conducted by Panizza et al.^(8)^, no skip lesions were observed.

## IMAGING EVALUATION

The imaging evaluation of PNS can be based on scans obtained with computed tomography (CT) or magnetic resonance imaging (MRI), the latter being much more sensitive, reaching a detection rate of up to 95%^(9)^. The technique is of paramount importance, and thin, high-resolution slices should always be acquired, in both methods. In MRI, volumetric T1- and T2-weighted sequences should always be obtained, as should volumetric contrast-enhanced T1-weighted sequences with fat suppression. It is believed that many cases of PNS go undetected because inappropriate techniques were employed. Even in asymptomatic patients, the entire nerve should be evaluated, from its terminal branches to its origin in the central nervous system.

The main findings that suggest PNS are enlargement and enhancement of the nerves, as well as of the foramina, or channels, through which the affected nerves pass, with consequent obliteration of fat planes. Indirect findings include denervation of muscles, whose first manifestation on MRI is as a hyperintense signal on T2-weighted images. Subsequently, there is a hyperintense signal on T1-weighted sequences and hypodensity on CT, due to liposubstitution and volume loss. Key points in the evaluation of each nerve specifically include the following:

V1 (ophthalmic branch of the trigeminal nerve) – Obliteration of the orbital fat pad along the bottom contour of the orbit, superior to the elevating muscle of the upper eyelid and orbital apex; thickening and enhancement of V1 in the orbit and the cavernous sinus.
V2 (maxillary branch of the trigeminal nerve) – Obliteration of the fat pads in the pre-antral region and pterygopalatine fossa; enlargement and erosion of the infraorbital fissure, as well as of the infraorbital and round foramina; thickening and enhancement of V2 in the round foramen and cavernous sinus.
V3 (mandibular branch of the trigeminal nerve) – Obliteration of the fat pads of the mental or mandibular foramen and of the parapharyngeal fat below the foramen ovale; enlargement or erosion of those foramina; thickening and enhancement of V3 in the parapharyngeal space and foramen ovale; abnormal bone marrow in the jaw; signs of denervation of the masticatory muscles, particularly the pterygoid, masseter, and superficial temporal muscles.
V (trigeminal nerve) – Blurring or obliteration of Meckel’s cave; thickening and enhancement of the trigeminal nerve.
VII (facial nerve) – Obliteration of the fat pad of the stylomastoid foramen; enlargement or erosion of the facial canal in the temporal bone.
Geniculate ganglion – Enlargement and obliteration of the geniculate fossa; bone sclerosis around the fossa and asymmetric enhancement in the geniculate fossa.
Greater superficial petrosal nerve – Obliteration of the fat pad in the pterygopalatine fossa and vidian canal; enlargement or erosion of the vidian canal.
Auriculotemporal nerve – Tumor growth in the posterior mandible; signs of dysfunction or pain in the temporomandibular joint.

## CASE ANALYSIS

**Figure 1:**
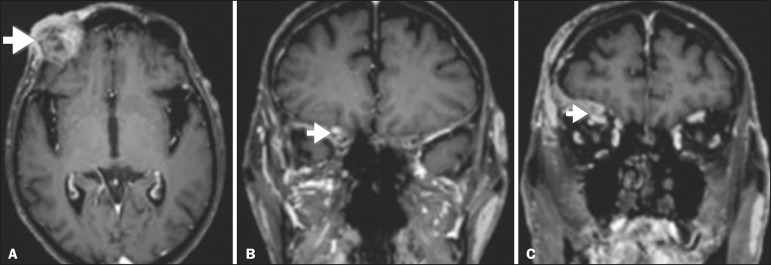
Contrast-enhanced T1-weighted MRI scans of a 83-year-old male patient with basocellular carcinoma (arrow in A), spread along the frontal nerve (B), V1, and supraorbital nerve (C), all of which are enlarged and enhanced.

**Figure 2:**
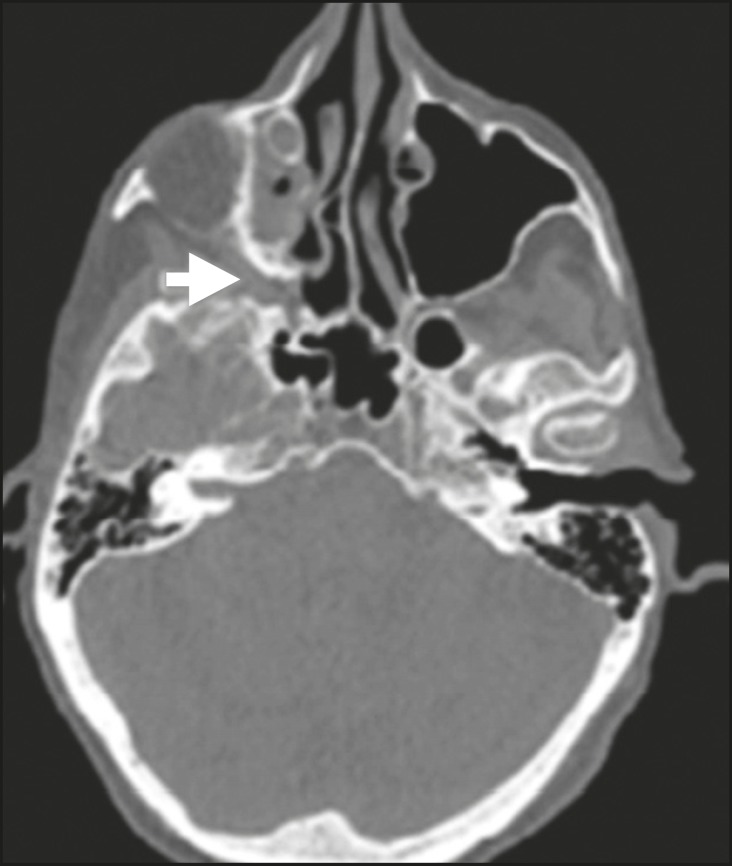
Contrast-enhanced CT scan of a 90- year-old female with squamous cell carcinoma of the maxilla showing enlargement and osteo­sclerosis of the right pterygopalatine fossa.

**Figure 3:**
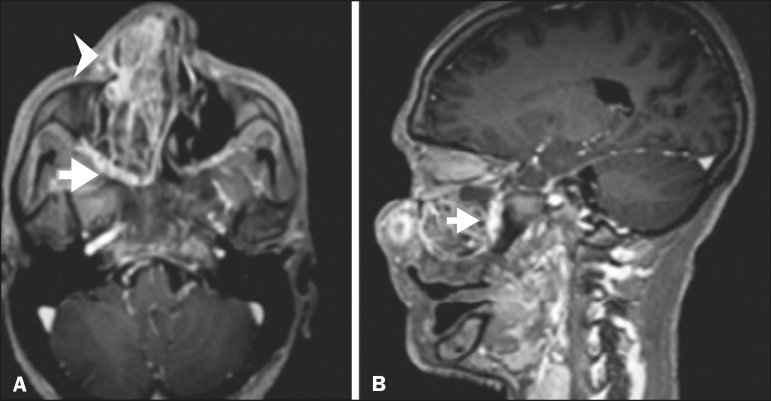
Gadolinium contrast-enhanced T1-weighted MRI scans of a 66-year-old female with squamous cell carcinoma spreading throughout the pterygopalatine fossa (arrows in A and B). The primary lesion is seen in A (arrowhead).

**Figure 4:**
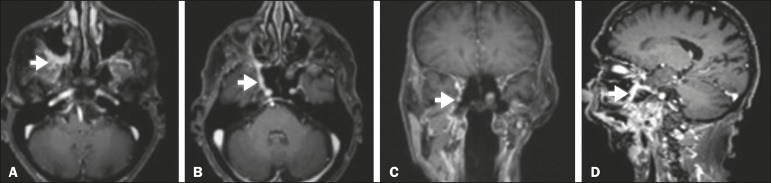
Gadolinium contrast-enhanced T1-weighted MRI scans of a 75-year-old man with buccal squamous cell carcinoma spreading throughout the right pterygopalatine fossa (arrows in A and D) and foramen rotundum (B and C).

**Figure 5:**
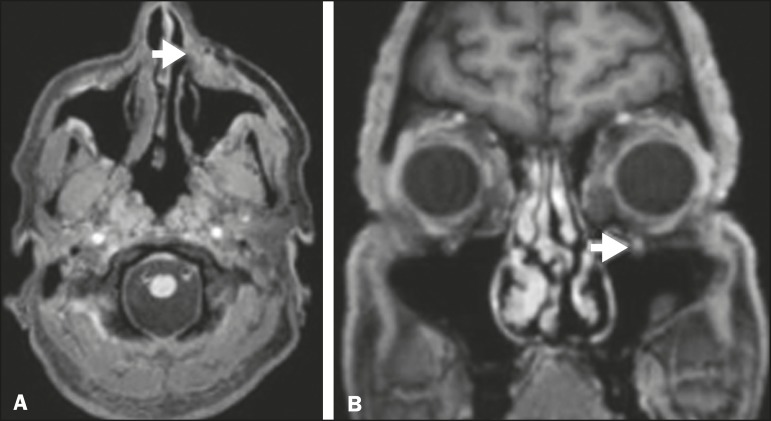
Gadolinium contrast-enhanced T1-weighted MRI scans of a 58-year-old man with basocellular carcinoma of the left nasal ala (arrow in A) spreading along the left infraorbital nerve, which is a branch of V2 (arrow in B).

**Figure 6:**
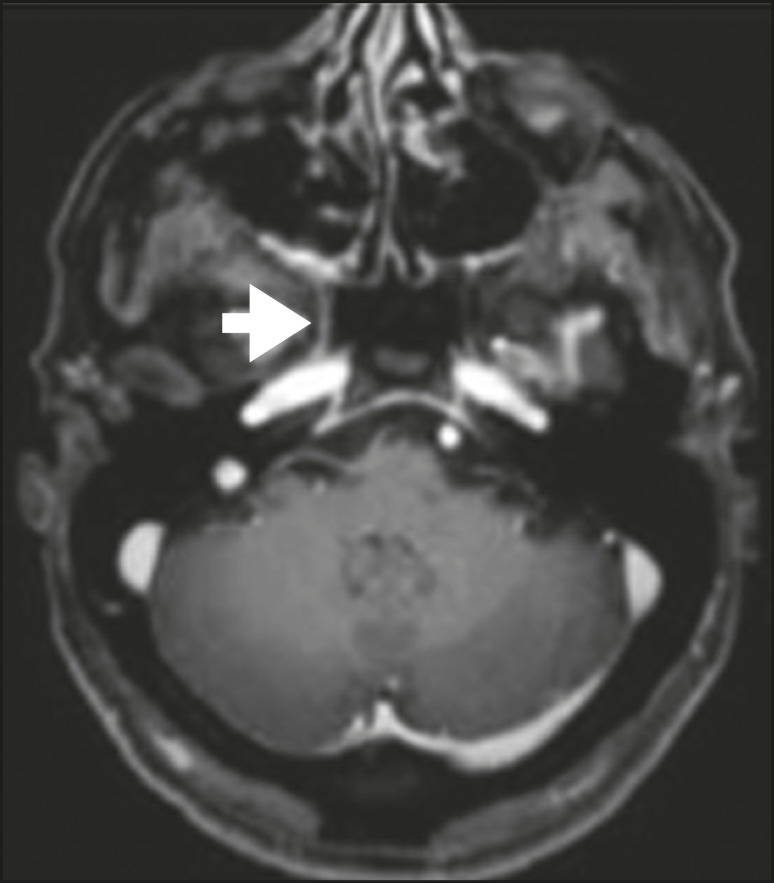
Gadolinium contrast-enhanced T1-weighted MRI scan of a 45-year-old male with polymorphic adenocarcinoma of the salivary gland spreading along the vidian nerve (arrow).

**Figure 7:**
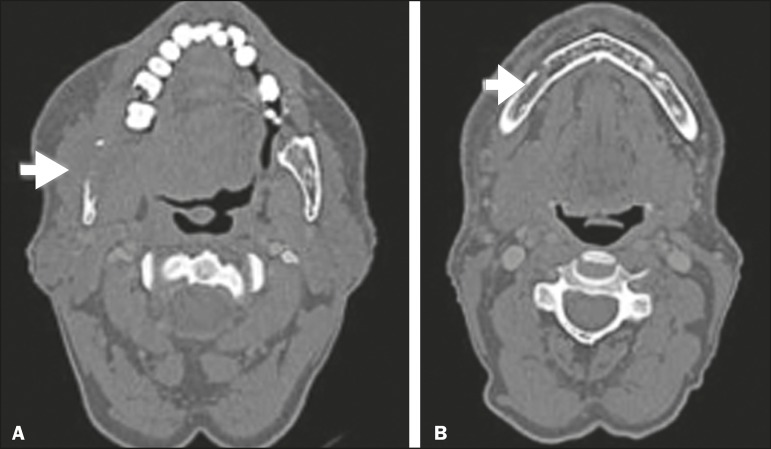
Contrast-enhanced CT scan of a 63-year-old male with squamous cell carcinoma of the right mandibular region, as depicted in image A, spreading along the inferior alveolar nerve (B). Note the enlargement of the mental foramen in comparison with the contralateral side.

**Figure 8:**
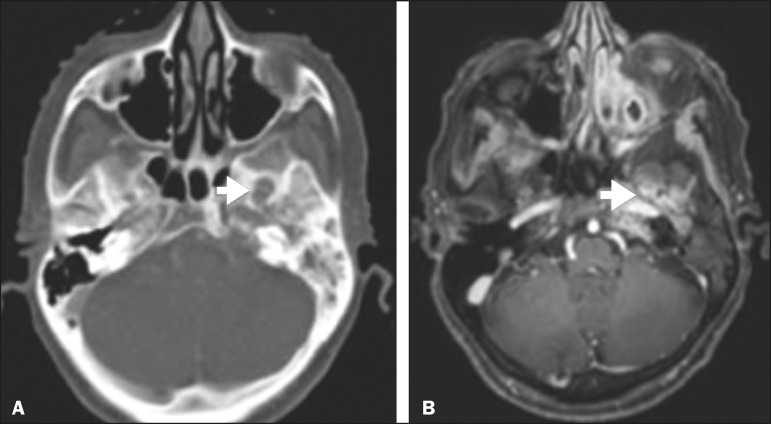
Contrast-enhanced CT (A) and gado­linium contrast-enhanced (B) T1-weighted MRI scans of a 63-year-old female with adenoid cystic carcinoma of salivary gland, depicting enlargement of the left foramen ovale and enhancement of V3, consistent with PNS.

**Figure 9:**
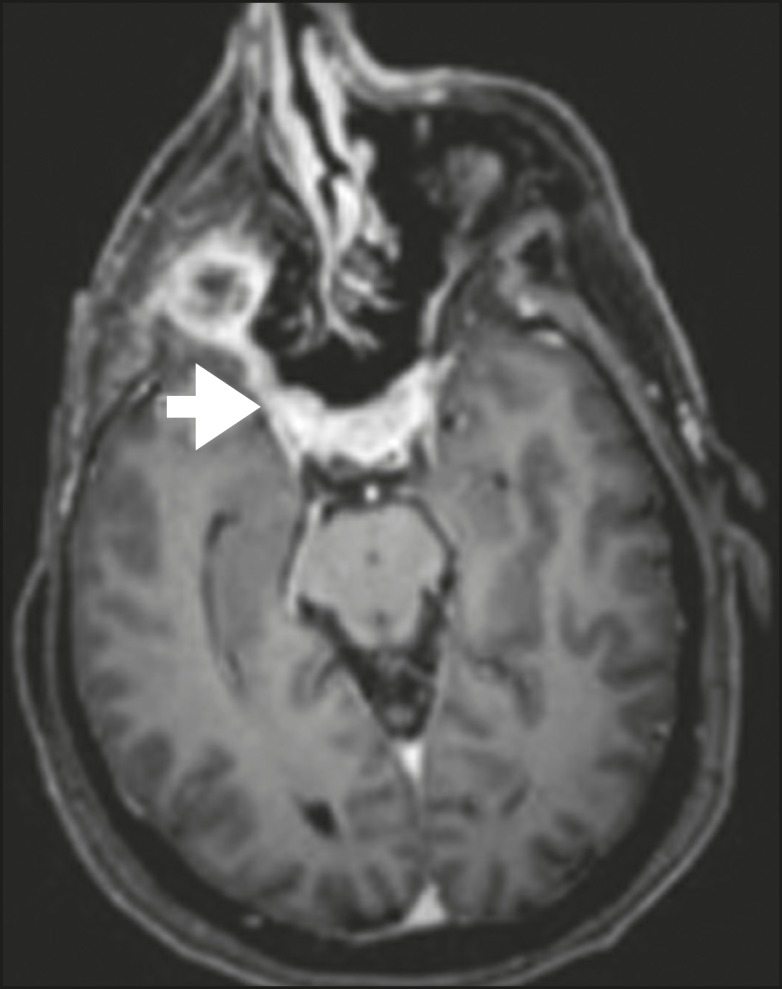
T1-weighted MRI scan of a 59-year-old male with (postoperative) squamous cell carcinoma and cervical metastasis. Note the spread along V2 (arrow).

**Figure 10:**
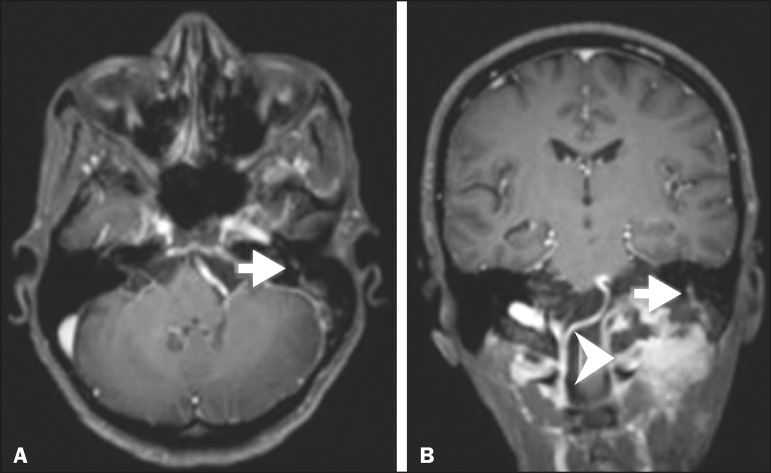
T1-weighted MRI scans of a 57-year-old female with (postoperative) squamous cell carcinoma spreading along the left facial nerve (arrows in A and B). The neoplasm can be seen in B (arrowhead).

The imaging tests of histologically confirmed cases of head and neck malignancies treated at the HCFMRP-USP were reviewed by two experienced radiologists, and ten cases with radiological signs of PNS were selected. The imaging tests were performed between 2006 and 2012. The radiological findings of PNS were described, and the data were analyzed. Seven patients underwent only MRI of the head in a 3.0 T scanner (Achieva; Philips Healthcare, Best, The Netherlands) with a specific head coil; two underwent only 16-channel multislice CT (Brilliance; Philips Healthcare); and one underwent both tests. The most prevalent tumor was found to be squamous cell carcinoma (60%), followed by basocellular carcinoma (20%), adenocarcinoma (10%), and adenoid cystic carcinoma (10%). The nerves most often affected were V2 and V3, followed by V1 and VII. The imaging findings are described below the images (Figures 1–10).

## CONCLUSION

PNS is an important parameter to be evaluated when staging patients with head and neck malignancies. Nevertheless, it is commonly overlooked in imaging tests, unless there is an active search by the radiologist. State-of-the-art techniques and intensive training of personnel are needed in order to increase the detection rate of this type of dissemination.
